# Supplementary material for: Na+ influx via Orai1 inhibits intracellular ATP-induced mTORC2 signaling to disrupt CD4 T cell gene expression and differentiation
Source: eLife. 2017 May 11;6:e25155. doi: 10.7554/eLife.25155 (PMC5459575; doi:10.7554/eLife.25155)
Supplement: Figure 6—source data 1. — Gene expression data from Figure 6G were subjected to pathway analysis and top 50 pathways that met the criteria described in Materials and methods are shown. DOI: http://dx.doi.org/10.7554/eLife.25155.011 [file elife-25155-fig6-data1.pdf]

Figure 6 -source data 1

Enrichment by Pathway Maps		
**Number**	**Maps**	**p-value**
1	Immune response_Generation of memory CD4+ T cells	7.72E-08
2	Development_Cytokine-mediated regulation of megakaryopoiesis	3.92E-07
3	Immune response_Differentiation of natural regulatory T cells	9.28E-06
4	Immune response_CD40 signaling	1.16E-05
5	Apoptosis and survival_Anti-apoptotic TNFs/NF-kB/Bcl-2 pathway	3.27E-05
6	Immune response_CD137 signaling in immune cell	3.38E-05
7	Transport_RAN regulation pathway	3.41E-05
8	Apoptosis and survival_Granzyme A signaling	4.14E-05
9	Immune response_CRTH2 signaling in Th2 cells	4.47E-05
10	Signal transduction_PTMs in IL-23 signaling pathway	5.04E-05
11	Cell cycle_Role of Nek in cell cycle regulation	6.09E-05
12	Immune response_IL-2 activation and signaling pathway	9.12E-05
13	Development_PEDF signaling	9.12E-05
14	Development_G-CSF signaling	9.12E-05
15	Immune response_IL-15 signaling via JAK-STAT cascade	1.23E-04
16	Immune response_IFN alpha/beta signaling pathway	1.52E-04
17	Immune response_Th17, Th22 and Th9 cell differentiation	1.93E-04
18	Immune response_Th1 and Th2 cell differentiation	2.23E-04
19	Transcription_Epigenetic regulation of gene expression	2.42E-04
20	Immune response_CCR5 signaling in macrophages and T lymphocytes	2.70E-04
21	Immune response_IL-10 signaling pathway	4.10E-04
22	Immune response_IL-4 - antiapoptotic action	4.58E-04
23	Immune response_Naive CD4+ T cell differentiation	4.87E-04
24	Immune response_IL-15 signaling	4.99E-04
25	Apoptosis and survival_Regulation of Apoptosis by Mitochondrial Proteins	7.22E-04
26	Development_GM-CSF signaling	7.68E-04
27	PDE4 regulation of cyto/chemokine expression in inflammatory skin diseases	7.68E-04
28	Translation_IL-2 regulation of translation	8.52E-04
29	Development_Regulation of telomere length and cellular immortalization	9.53E-04
30	Immune response_IL-9 signaling pathway	1.09E-03
31	Immune response_IL-16 signaling pathway	1.16E-03
32	Development_Thrombopoetin signaling via JAK-STAT pathway	1.24E-03
33	Apoptosis and survival_APRIL and BAFF signaling	1.57E-03
34	Immune response_IL-27 signaling pathway	1.74E-03
35	Immune response_IL-17 signaling pathways	2.01E-03
36	Immune response_IL-18 signaling	2.01E-03
37	Immune response_IL-23 signaling pathway	2.04E-03
38	Apoptosis and survival_Lymphotoxin-beta receptor signaling	2.20E-03
39	Immune response_IL-7 signaling in B lymphocytes	2.45E-03
40	Signal transduction_AKT signaling	2.45E-03
41	Immune response_IL-4 signaling pathway	2.71E-03
42	Immune response_TNF-R2 signaling pathways	3.00E-03
43	Immune response_NF-AT signaling and leukocyte interactions	3.63E-03
44	Immune response_TLR5, TLR7, TLR8 and TLR9 signaling pathways	3.99E-03
45	Development_TGF-beta receptor signaling	4.76E-03
46	Immune response_T regulatory cell-mediated modulation of effector T cell and NK cell functions	6.11E-03
47	ATP/ITP metabolism	6.15E-03
48	Development_S1P1 receptor signaling via beta-arrestin	6.41E-03
49	Immune response_IL-22 signaling pathway	6.41E-03
50	Immune response _IFN gamma signaling pathway	6.62E-03
